# Scoring of protein–protein docking models utilizing predicted interface residues

**DOI:** 10.1002/prot.26330

**Published:** 2022-03-14

**Authors:** Gabriele Pozzati, Petras Kundrotas, Arne Elofsson

**Affiliations:** ^1^ Department of Biochemistry and Biophysics and Science for Life Laboratory Stockholm University Solna Sweden; ^2^ Center for Bioinformatics and Department of Molecular Biosciences University of Kansas Lawrence Kansas USA

**Keywords:** protein bioinformatics, protein docking, protein interaction predictions, protein structure predictions, protein–protein interactions

## Abstract

Scoring docking solutions is a difficult task, and many methods have been developed for this purpose. In docking, only a handful of the hundreds of thousands of models generated by docking algorithms are acceptable, causing difficulties when developing scoring functions. Today's best scoring functions can significantly increase the number of top‐ranked models but still fail for most targets. Here, we examine the possibility of utilizing predicted interface residues to score docking models generated during the scan stage of a docking algorithm. Many methods have been developed to infer the regions of a protein surface that interact with another protein, but most have not been benchmarked using docking algorithms. This study systematically tests different interface prediction methods for scoring >300.000 low‐resolution rigid‐body template free docking decoys. Overall we find that contact‐based interface prediction by BIPSPI is the best method to score docking solutions, with >12% of first ranked docking models being acceptable. Additional experiments indicated precision as a high‐importance metric when estimating interface prediction quality, focusing on docking constraints production. Finally, we discussed several limitations for adopting interface predictions as constraints in a docking protocol.

## 1 INTRODUCTION

Most proteins carry out their biological functions through interactions with other proteins.^1^ Subsequently, the ability to modulate protein–protein interactions (PPI) could lead, among other things, to the cure of diseases. However, modulating PPIs requires a fundamental understanding of PPI details on the atomic level. Experimental methods, like X‐ray crystallography or NMR/EM spectroscopy, can produce highly reliable structures, but unfortunately, these methods are expensive and time‐consuming.^2^

A completely different approach to derive such structures involves using computational methods.^3^ Unfortunately, this approach is limited by the dynamic nature of protein behavior in vivo. For instance, most proteins undergo structural rearrangements or conformational changes when interacting with a partner.^4^ Also, in some cases, PPI is obligate, meaning that the protein must fold into a stable and functional conformation.^4^, ^5^ Other PPIs are nonobligate, meaning interaction partners may also exist in a stable but nonassociated form. Obligated complexes are generally permanent, but most nonobligate complexes are transient. Their lifetime is influenced by several factors, including physiological conditions (pH, salt concentration, etc.), the concentration of interaction partners, and the state of certain molecular switches.^5^ Furthermore, obligate and nonobligate complexes have different geometrical and physicochemical properties of their interfaces.^6^ Thus, the prediction of three dimensional structures of protein–protein complexes (protein docking) remains one of the most demanding challenges in computational biology.

Usually, a structure of uncharacterized PPI is derived from structures (experimental or modeled) of individual proteins by rigid‐body^7^ or flexible docking procedures.^8^, ^9^ These protocols generally consist of two stages: fast generation of large numbers of putative mutual arrangements of two proteins (docking model or pose) using simplified energy function (scan stage) and subsequent application of a more complex scoring function to the obtained configurations to discriminate the few ones that most likely are close to the native structure (scoring stage).^10^ Rigid‐body docking is generally faster than flexible docking. Still flexible docking (that allows intraprotein conformational degrees of freedom) better reflects the dynamic nature of the proteins.^9^ Limitations of these methods are implicit in the necessity to generate large amounts of the docking models (usually on the order of hundreds of thousands) to have a significant chance of generating at least one near‐native docking model. Many decoys are not a problem but necessitate an extraordinarily accurate and computationally efficient method to identify the few near‐native solutions. Some methods also use much smaller datasets for testing,^11^ that is, these methods do not work for the general docking problem. Another common strategy is reducing the number of considered docking poses by performing clustering and only applying a scoring function to the cluster representatives.^10^ With such an approach, acceptable docking models can be found in the top 10 scored poses for almost 40% of complexes in the widely adopted Benchmark 5.0 dataset.^12^, ^13^

Another approach is to use constraints derived from predicting which residues from the surface of one protein are more likely to belong to the interaction interface but without specifying individual contacts (interface prediction).^14^ Constraint generation can also be done by predicting specific pairs of residues from different chains that are closer than a threshold distance (contact prediction).^15^ In recent years, many interface‐ and contact‐prediction algorithms have been published.^16^, ^17^, ^18^, ^19^, ^20^, ^21^, ^22^, ^23^, ^24^, ^25^ However, without testing how they would improve the success of protein docking algorithms. Most predictors use different combinations of sequence and structural features of proteins in their unbound (interacting interface completely exposed to the solvent) and bound (associated) forms^16^, ^20^, ^26^ along with the evolutionary features acquired from the standard multiple sequence alignments (MSA).^15^, ^27^ In general, contact prediction is harder compared to interface prediction.^28^ On the other hand, using single‐protein MSA in the interface prediction is advantageous as combining MSAs from different interacting proteins (required for some interface contact prediction algorithms) is a nontrivial task.^29^ Another main advantage of predicting interface patches is that, considering proteins singularly equalize on a similar order of magnitudes, the number of interacting and noninteracting residues, making the two categories more or less balanced, according to the protein type. This last property is important for all the machine learning methods commonly applied to this problem, particularly support vector machines (SVMs) and artificial neural networks (ANNs). Indeed, most machine learning algorithms are consistently influenced by unbalanced datasets and tend to learn undesired patterns, such as proportions of classes, from the provided trainingset.^30^

Dockrank is one of the most recent attempts to use interface predictions in protein–protein docking.^31^ This work has shown some consistent improvement in the docking success when applying interface predictions to the scoring of the docking poses. However, the dataset used in that study was limited to complexes with sufficient confidence of predicted interface residues, which reduces the generalization of the conclusions. Furthermore, other studies were conducted on small or bound datasets only, and in some cases, the predicted interface information was used in combination with other scoring parameters, which made the exact contribution of interface predictions unclear.^32^, ^33^, ^34^ Thus, it is still unclear how much valuable information for docking can be extracted from interface prediction. In order to clarify this point, we filter docking poses produced by the GRAMM docking software,^35^ utilizing interface information acquired from native structures of PPI in the DOCKGROUND dataset and various interface predictors. This protocol aims to establish a reference framework for easy quantification of the performance of different interface predictors when applying them in a real‐case docking scenario when the native PPI structure is not known.

## 2 MATERIALS AND METHODS

### 2.1 Dataset

This study utilized all dimeric protein complexes extracted from the benchmark set 4^36^ from the Unbound section in the DOCKGROUND website: http://dockground.bioinformatics.ku.edu/. Additionally, we excluded all the complexes containing chains shorter than 50 residues, leading to a set of 220 protein pairs for which both single‐chain (unbound) and associated (bound) experimental structures are available. Finally, we clustered all sequences against all sequences from the Benchmark 5 dataset^12^ at 20% sequence identity using the CD‐hit software^37^ (version: 4.7). This allowed us to exclude dimers when both chains had a higher than 20% identity to any Benchmark 5 entry, retaining in total 175 dimers. Depending on root‐mean‐square deviation (RMSD) between interface Ca atoms in unbound and bound structures (*i*‐RMSD) and fraction of non‐native contacts (fnon‐nat) in unbound structures,^12^ this dataset can be divided into 98 easy (*i*‐RMSD <1.5 Å and fnon‐nat <0.4), 51 medium‐difficulty (1.5 < *i*‐RMSD < 2.2 and fnon‐nat >0.4), and 26 hard (i‐RMSD >2.2) cases.

The numbering of residues in the unbound structures has been mapped to the numbering in the bound structures using pairwise global sequence alignment utility from the biopython package (version: 1.76) with the BLOSUM62 scoring matrix.^38^ In order to facilitate the following comparisons, all residues from bound structures with no correspondence in the unbound structures have been trimmed. Furthermore, unbound chains have been structurally aligned to the bound counterpart to determine a level of difficulty for the docking of each complex. Here we adopted three difficulty classes; hard, medium, and easy, as described previously.^39^ Finally, for each complex, the longer (shorter) chain has been re‐labeled “A” (“B”) and henceforth is referred to as receptor (ligand).

The same re‐numbering and chain re‐labeling scheme has been applied to dimeric targets selected from the CAPRI Score_set^40^ to obtain an additional testing set. From this sub‐set, we excluded T36 which is identical to T35, T39, which is identical to T38 and T47, whose unbound chains are identical to the bound chains of T41. Also, we excluded T29 due to the impossibility to obtain predictions from the Predus2 web server for that specific target. This resulted in eight heterodimeric complexes for which bound and unbound configurations are available. PDB codes for this set are summarized in Table S1.

### 2.2 Rigid‐body docking protocol

Unbound structures of the proteins in the dataset were docked utilizing Fast Fourier transform (FFT) rigid‐body docking algorithm as implemented in the scan stage of the GRAMM software.^35^ Unlike other FFT‐based programs (e.g., ZDOCK^7^ and ClusPro^41^), GRAMM does not include any other energy components (electrostatics, desolvation, etc.) besides simplified Lennard–Jones potential when generating an initial set of docking poses. Therefore, using all these models allows investigating the “pure” effect of various factors on a minimally biased set of docking models generated with only the surface geometry of the receptor and ligand taken into account. Further, the unique low‐resolution nature of the GRAMM docking algorithm permits small amounts of atomic clashes on the interfaces of the docking models, which to a certain degree accounts for the conformational flexibility upon protein binding.^35^

Default grid sizes (32 × 32 × 32 or 64 × 64 × 64) and calculation parameters (grid step 3.5 Å, rotation angle 10°) have been used for all complexes except 4YOC, where it was necessary to increase grid size to 128 × 128 × 128. To ensure that at least one near‐native docking model is presented for all the complexes considered, 340 000 docking poses were generated for each docking pair. GRAMM output (translation vector and three Euler angles per docking pose) were transformed into Cartesian coordinates of the ligand using a script written with the Tensorflow python library (version: 1.13.2). Different dockings may be elaborated in parallel in both steps, consistently reducing the computation time. The initial docking poses were further re‐scored by using the following function:

(1) $$S=\sum_{i=1}^{N_{1}}\sum_{j=1}^{N_{2}}\left\{\begin{array}{cc} -\ln\left(1-\left(p_{i}*p_{j}\right)\right), & \text{if}\;d_{\mathrm{ij}}<12Å\\ 0 & \text{otherwise} \end{array}\right.$$

where the summation is performed over all *N*_1_ and *N*_2_ residues of the receptor and ligand, respectively, *p*_*i*_ and *p*_*j*_ are, correspondingly, the probabilities (given by an interface predictor) of residues *i* of the receptor and *j* of the ligand to occur on the native interface, where *d*_*ij*_ is the distance between *C*_*β*_ atoms of residue *i* in the receptor and residue *j* in the ligand. The 12 Å distance threshold has been considered according to what is established by Sinha et al.^42^ and adopted, after proper testing of different options (8, 10, and 12 Å), as the optimal interface threshold for scoring docking solutions. In order to avoid singularities in Equation (1), an upper limit of 0.99 for *p*_i_ and *p*_*j*_ was used. Ten highly‐scoring docking poses were retained for further evaluation. We also used docking poses re‐scored by the atom‐atom contact energy AACE18^43^ for comparison.

### 2.3 Alternative docking protocol

In order to test scoring of the docking poses with interface predictions with a different docking approach, we utilized the LzerD server,^44^ which was ranked second in the server part of the CASP14‐CAPRI experiment, is available both as a web‐server and as a standalone version, and accepts user‐specified restraints. In this study, we ran the eight complexes from the CAPRI Score_Set on the web‐server with restraints consisting of 10 interface predictions with the highest probability for each protein chain (20 restraints for each dimer). In case of categorical predictors, where no probabilities are available, 10 random residues predicted to occur in the interface have been selected instead. Such restraints have been formatted in a JSON file, specifying that for each indicated residue a relaxed distance between 2 and 8 Å from the residue to the partner chain must be matched. We required that in the docking models at least 5 out of 10 restraints should be satisfied.

### 2.4 Interface predictions

We selected several predictors (Table 1) for calculating propensities of the residues to occur inside the native interaction patch. We are aware that there are many more interface predictors described in the literature, but our choice was restricted by the availability and portability of the code to run locally. BIPSPI^19^ produces estimates of interface patches from predicted interprotein contacts for a pair of either sequences or structures. In this study, pairs of structures were provided as input, and the two interfaces returned from the predictor were used for scoring. ISPRED4^26^ first uses a SVM to generate initial interface residue propensities. In ISPRED4, these predictions are further processed by conditional random fields (CRFs). However, no improvement was seen in our study using the second set and, therefore, the CRF predictions were ignored.

**TABLE 1:** Interface residue predictors

Predictor	Description	References
SPPIDER	Neural network consensus method based on protein structure geometric features and predictions of relative solvent accessibility.	[45]
PredUs2	Support vector machine method based on solvent accessibility and position conservation derived from protein structural alignment.	[46]
dynJET2	A model combines evolutionary, geometric, physicochemical, and interface propensity features.	[47]
ISPRED4	A method based on support vector machine and conditional random fields combines residue structural context, physicochemical, and multiple sequence alignment features.	[26]
BIPSPI	Tree classifier trained with XGBoost algorithm, based on structural and multiple sequence alignment features obtained for pairs of proteins.	[19]
DeepInteract	Geometric transformer deep‐learning model based on structural and evolutionary (nonpaired) features.	[48]
RaptorX ComplexContact	Deep residual neural‐network method relying on phylogeny‐based MSA‐pairing.	[24]
trRosetta	Deep residual neural‐network, relying on paired MSA information.	[25]

Further, SVM‐based binary interface predictions have also been obtained using the PredUS predictor.^46^ The dynJET2 algorithm^47^ has been applied to our dataset with the automatic mode selection option (−a 0), using 10 iterations as suggested by the authors. Each residue has been conducted to belong to an interface supported by two or more clustering iterations, setting the probability to zero otherwise. The SPPIDERII algorithm from the SPPIDER Web server [33] was used to generate predictions in the regression form, obtaining continuous probabilities from 0 to 1 (all other options have been left at their default values).

Deepinteract is a recent interprotein contact prediction method that utilizes a neural network with a geometric transformer.^48^ RaptorX Complex‐Contact^24^ is a sequence‐based contact prediction method that has been adopted through a web‐server. Finally, we also applied trRosetta,^25^ a method to predict intrachain residue‐residue distances, which has also been successfully applied to interchain predictions in our previous work.^49^ In order to obtain a common comparison ground with interface predictions, in the case of Deepinteract and RaptorX‐ComplexContact, we selected the highest contact probability for each residue as a surrogate of an interface prediction. For the trRosetta, binned distance probabilities have been converted first to contact probabilities by computing the ratio of the first 20 bins sum (which match the 12 Å threshold) over the sum of all bins. Then, the maximum contact probability has been selected for each residue to represent the related interface prediction.

### 2.5 Native interfaces

Native interface residues were extracted from the bound structures using the condition that solvent accessible surface area (SASA) of a residue in a protein in isolation should be larger than when the protein is bound to the interacting partner. SASA was calculated employing the DSSP v.3.0.0 module^50^ implemented in the biopython library. If a residue from the unbound structure had no correspondence to the bound one, the same criteria were evaluated on unbound structures superimposed on the corresponding bound.

### 2.6 Assessment of interface predictors

Interface prediction quality has been evaluated using two classic metrics: True Positive Rate or Recall, TPR:

(2) $$\mathrm{TPR}=\frac{\mathrm{TP}}{\mathrm{TP}+\mathrm{FN}}$$

and Precision, PPV:

(3) $$\mathrm{PPV}=\frac{\mathrm{TP}}{\mathrm{TP}+\mathrm{FP}}$$

where TP, FP, and FN are the numbers of true positives (correctly predicted interface residues), false positives (noninterface residues incorrectly predicted as interface) and false negatives (interface residues incorrectly predicted as noninterface) for a specific protein chain. For the interface predictors that output continuous probabilities rather than binary classification (interface/noninterface), all those quantities are dependent on the probability threshold, above which a residue is considered to be on the interface. Thus, to evaluate the overall performance of such predictors, we used the area under the precision‐recall curve (AUC) computed for decreasing thresholds using the scikit‐learn python package (v. 0.24.1). In our pipeline, an interface predictor produces two predictions for each protein complex considered (one for receptor and another for a ligand) with generally different AUC. We use both sets per complex or a set with the smaller AUC (henceforth referred to as *worst chain* predictions) for further analysis. For evaluating the overall performance of an interface predictor, we averaged TPR and PPV values for all protein chains in the dataset and analyzed the distribution of AUC values.

### 2.7 Assessment of docking predictions

To assess the quality of a docking model, we adopted the DockQ score,^51^ which combines all evaluation criteria used in the CAPRI competition^52^ into a single score, into a range from 0 to 1, with 1 representing a perfect match between a docking model and the native complex structure. Here, DockQ values of 0.23, 0.49, and 0.8 represent threshold values^51^ for docking models of acceptable, medium, and high quality in terms of the CAPRI criteria. DockQ scores were obtained by comparing a docking model with the bound version of the complex structure if not specified differently. To measure the overall performance of a docking protocol over the entire dataset, we evaluated the fraction of acceptable models (defined by DockQ >0.23), *SR*(*N*), in the top *N* ranked models. Here, we analyzed *SR*(*N*) for all *N* ≤10.

### 2.8 Simulated interface predictions

To observe the behavior of interface prediction‐driven docking in a controlled scenario, simulated interface predictions have been generated by introducing predefined levels of noise in the native interfaces. First, randomly selected interface residues from each protein chain were marked as noninterface to reach a certain TPR. After that, randomly selected surface residues not belonging to the interface were marked as interface until reaching a certain value of PPV. In this study, we considered nine different datasets with various (TPR/PPV) values: (0.25/1), (0.5/1), (0.75/1), (1/0.25), (1/0.5), and (1/0.75).

### 2.9 Availability

All code is available from git https://github.com/ElofssonLab/BindingSite_docking. All data for all methods are available from https://figshare.com/s/1803e314859b537d1e72.

## 3 RESULTS AND DISCUSSIONS

### 3.1 Baselines for the docking performance

**FIGURE 1:**
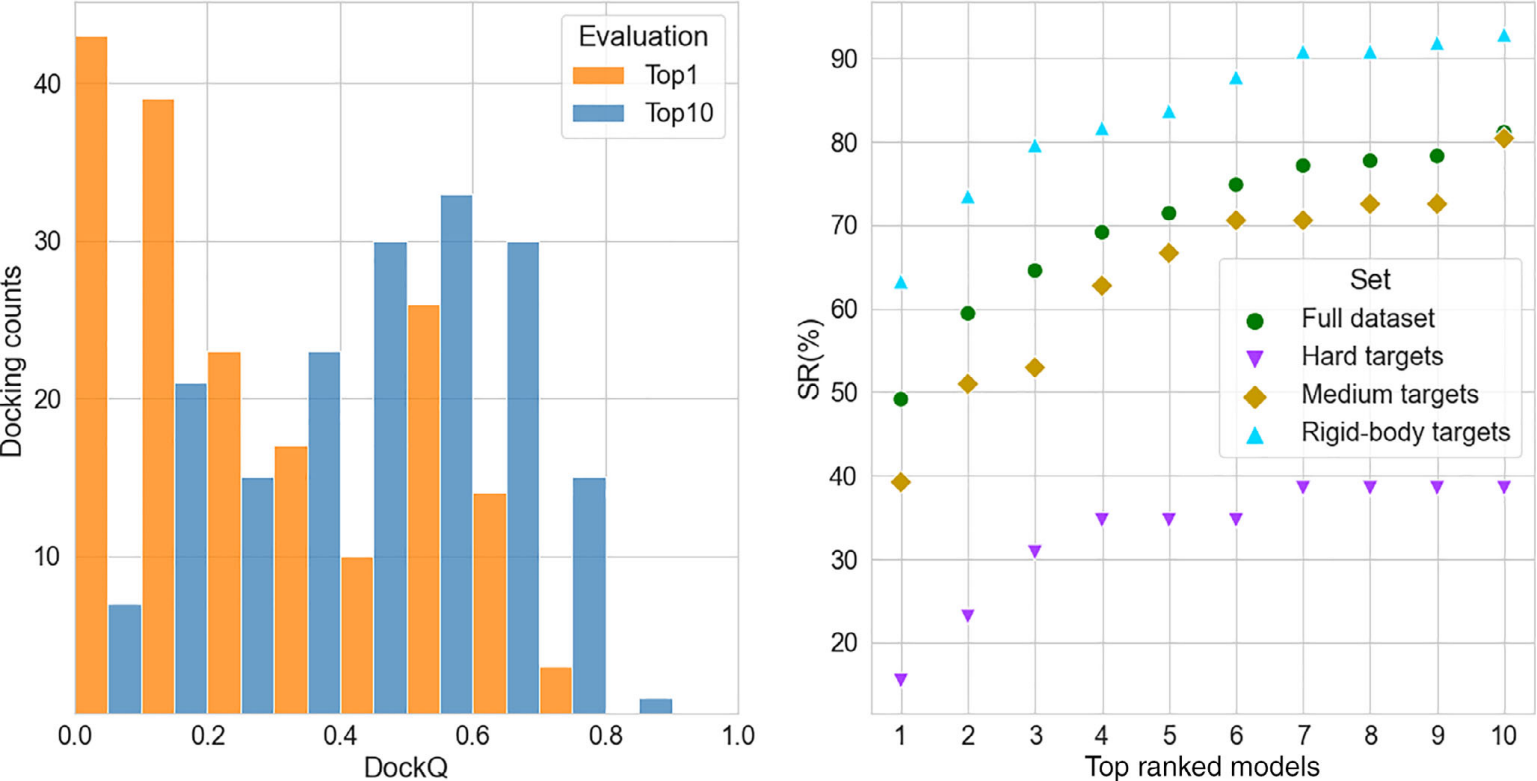
Performance of the docking with constraints derived from the native interfaces; (left) distribution of DockQ scores; (right) success rate, *SC*(*N*), as a function of the number, *N*, of considered top docking models. Data in the left panel pertains to the entire dataset of 220 binary complexes from DOCKGROUND benchmark set 4, while the right panel displays results for the entire dataset and the three sub‐groups separately

The lower baseline for our docking pipeline was determined by analyzing “raw” GRAMM output (ranked by shape complementarity only). Then, the docking protocol yielded at least one acceptable docking model among the top 10 models for 12 complexes (*SR*[10] ~5%) with an average DockQ score of 0.04. The upper baseline was estimated using all native interface residues by setting *p*_*i*_ and *p*_*j*_ in Equation (1) to a probability of 0.99. In this case, SR(10) jumps to 81%, with an average DockQ score of 0.45. Top ranking models are of acceptable or better quality for almost half of the targets, SR(1) ~49% with average DockQ ~0.27. While using the native interface residues as constraints, we tested different distance thresholds (8, 10, and 12 Å) to fit into Equation (1). This test allowed us to select 12 Å as the optimal value, given the higher SR(1) compared to 8 Å (SR(1) ~43%) and 10 Å (SR(1) ~42%) thresholds. Easy cases from the dataset yielded SR(1) ~ 62%, but even medium and hard cases displayed significant SR(1), with 35% and 16%, respectively (Figure 1, right panel).

Among the 42 targets with no acceptable docking models in the top 10 models, there are 7 easy, 18 medium‐difficulty, and 17 hard examples (6%, 25%, and 53% of corresponding cases in the entire dataset). The lower performance on the hard targets indicates the significance of accounting for the flexibility in the docking protocol. Nevertheless, near‐native docking models are present further down the list for all complexes in the dataset.

**FIGURE 2:**
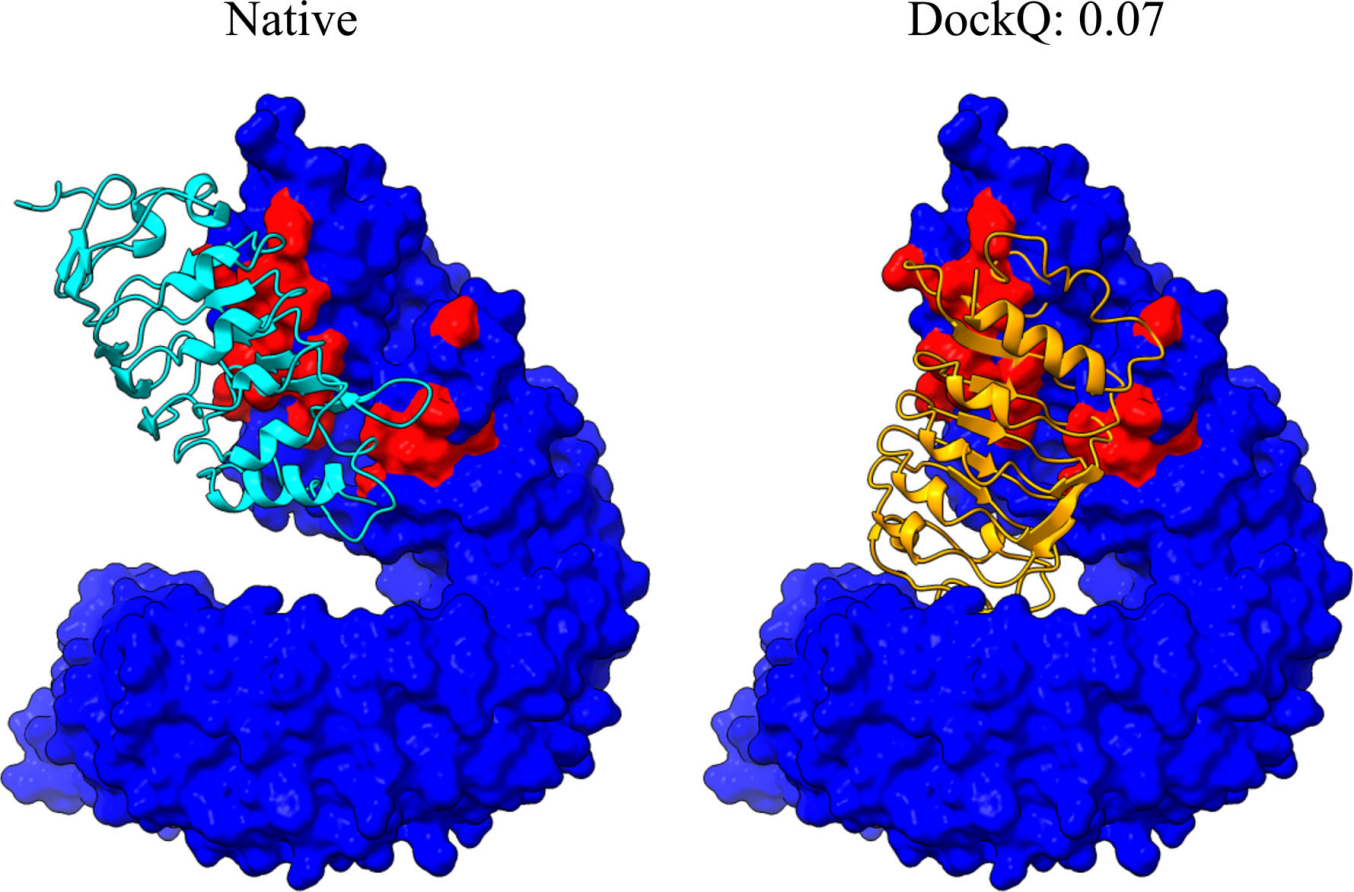
Example of the docking impaired by maximization of the number of contacts. The left panel displays a reference “native” complex built by the superimposition of the unbound structures taken from PDB 4LSA, chain A (receptor) and 4LSC, chain A (ligand) onto, correspondingly, the chains A and C of the PDB 4LSX. The right panel depicts the best docking model among the top 10 models re‐scored by equation (Equation 1) with the 99% probabilities for the native interface residues. In both panels, receptors are represented by the atomic surfaces and colored red (blue) for the native interface (noninterface) residues, while ligands are displayed as the cartoons

**FIGURE 3:**
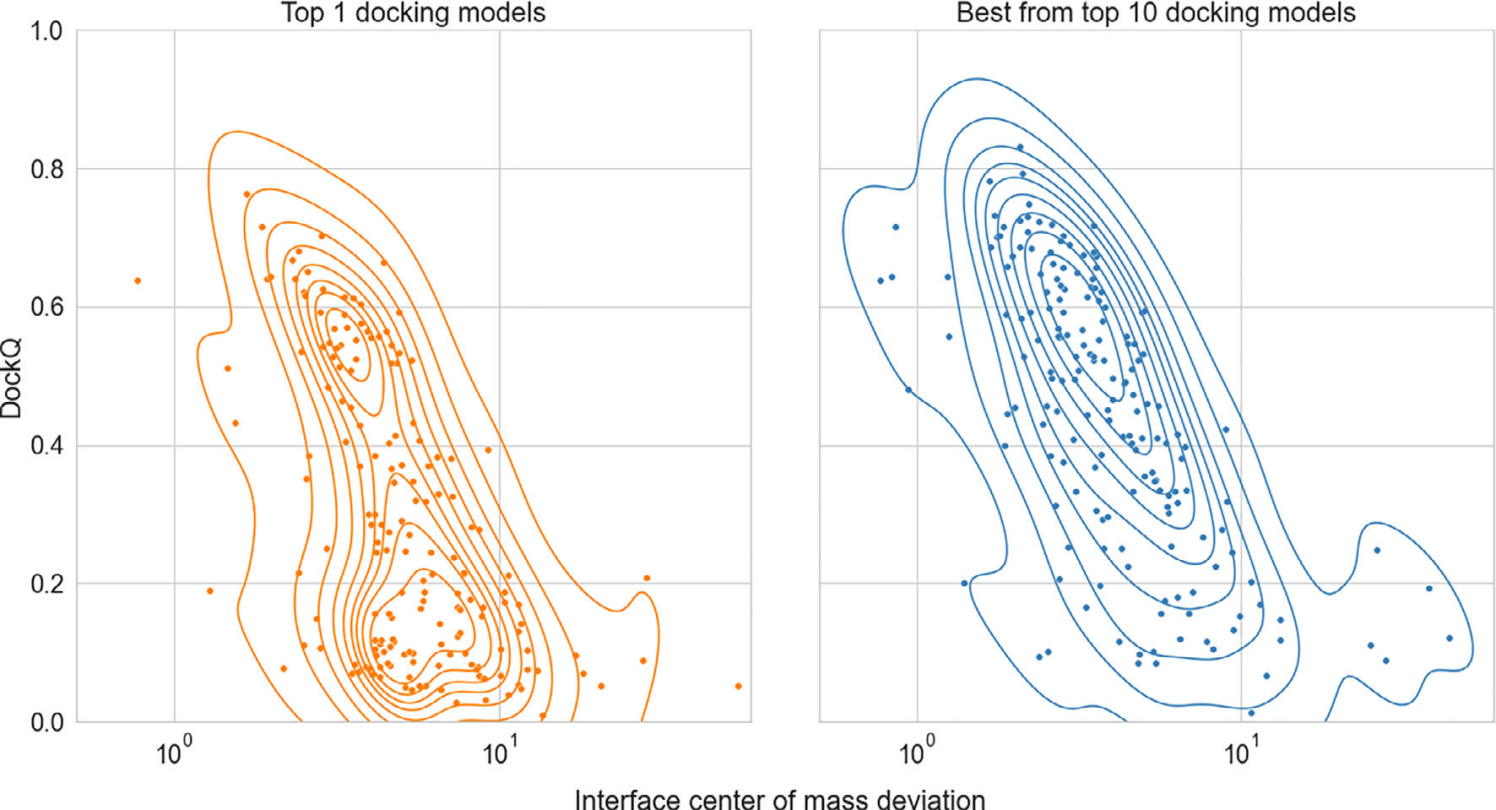
Interface center of mass deviation correlated to DockQ score from dockings with real interface constraints. For each complex in the DOCKGROUND benchmark‐4 dataset, the center of mass coordinates computed for the native complex interface residues and the identical residues in the best docking solution (in top rank and top 10 ranks) were obtained using real interface constraints. The distance between the two centers of mass has been plotted against the relative docking model DockQ score. Kernel density estimator (seaborn library, default settings) has been adopted to improve density visualization

However, the difference between bound and unbound conformations of the proteins in the dataset led in several cases to the imperfect shape complementarity in the unbound “native” PPI structure (unbound structures superimposed on the bound ones in their native arrangement) while scoring equation (Equation 1) favorises docking conformations with more contacts. In addition, docking constraints utilized in this study are considered on the residue level rather than on the residue contact level. Hence, the current re‐scoring scheme may bring to the top of the prediction list docking models that have interface patches of the receptor and ligand surfaces correctly facing each other, but with the ligand rotated so that this mutual ligand and receptor position maximizes the number of contacts for the unbound structures (an example is shown in Figure 2). Indeed, there is a significant number of top 1 docking models with a slight deviation of their interface center of mass (CM) from the CM of the native interface (Figure 3A). Notably, for the best out of the top 10 docking models, this number is significantly smaller, and the DockQ score exhibits the expected correlation with the CM deviation (Figure 3B), indicating that given correct interface constraints, it is desirable to analyze top 10 models in order to infer docking models with correct mutual orientation of the receptor and ligand.

### 3.2 Performance of interface predictors

**FIGURE 4:**
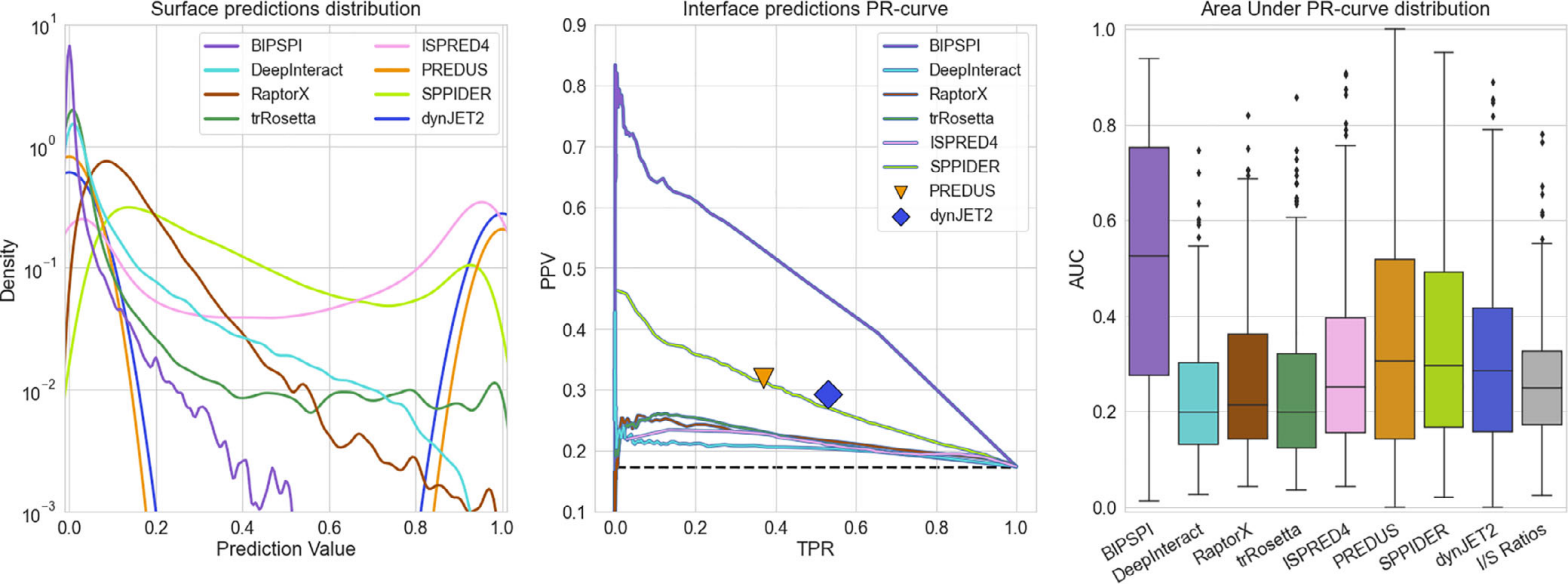
The overall behavior of different interface predictors on 220 binary complexes from DOCKGROUND benchmark set 4. In the left panel, the distribution of predictions for each surface amino acid in the dataset has been reported for each predictor in the left panel. The mid panel displays precision‐recall curves with TPR and PPV averaged over all protein chains in the dataset. The dashed line represents the average ratio of interface and surface residues (I/S ratio), which serves as the expected performance of a random predictor. For PREDUS and dynJET2, no AUC curve can be drawn; thus, single markers represent its performance. In the right panel, AUC distributions for individual protein chains are shown as Box and Whiskers plots, each corresponding to a different interface predictor. For PREDUS and dynJET2, PPV values are reported instead due to their equivalence to the AUC. As a random predictor reference, the distribution of the I/S ratios for individual protein chains is shown

The best overall identification of interface residues is observed for the BIPSPI predictor, with an AUC of 0.46 (Figure 4, left panel), clearly superior to the other methods (AUC: 0.20–0.32). Further, predictions from PredUS2 and DynJET2 have been evaluated using a single combination of TPR and PPV due to the binary output. These predictors reached a performance comparable to SPPIDER (AUC = 0.29), with TPR = 0.37, 0.53 and PPV = 0.32, 0.29 for PredUS2 and DynJET2, respectively. A large performance gap can be observed with different contact prediction methods. Deepinteract, RaptorX, and trRosetta yield the lowest performance with ISPRED4 (AUC = 0.20–0.22). Examining the overall distribution of individual chains, all predictors, except BIPSPI, have similar median values ranging between 0.20 and 0.31. However, the number of chains being predicted better than random varies widely, from 25% of Deepinteract to 77% of BIPSPI. When the performance of interface predictors are assessed using *worst chain* predictions (see Section 2), the precision‐recall curves obtained a behavior very similar to what was expected from a random predictor (AUC = 0.2), data not shown. Again, the only exception is BIPSPI, which yielded an average AUC of 0.32.

Notably, BIPSPI is the only predictor that considers pairs of structures simultaneously to infer their interface. All the other interface predictors use only a single structure. Therefore, they might predict alternative interfaces, interacting with different interaction partners, possibly explaining the superior performances of BIPSPI. RaptorX and trRosetta consider pairs of sequence‐derived features as input but make no use of structural information, which appears to be a consistent limitation in protein docking. The most similar method to BIPSPI is Deepinteract, which differs only in the final output. Further, all predictors, except BIPSPI,^19^ consistently perform worse than reported in the original publications. The decreased performance could be related to overtraining of the methods.

One indirect confirmation for this hypothesis is given by studying the structural similarity of complexes with 0.25 Interface‐Surface ratio (Figure S1, right panel) to the complexes from the original BIPSPI training set (Benchmark 5^12^), which are responsible for a consistent peak in interface prediction AUC. The average TM‐score for this set is 0.89. In comparison, the complexes responsible for the drop in AUC at I/S ratio 0.29 (Figure S1, right panel) have an average TM‐score of only 0.59. To further verify this, each complex TM‐score has been compared with the worst interface predictions derived from BIPSPI (Figure S2, left panel). This comparison displayed a spearman correlation coefficient of 0.48 between training set similarity and interface prediction performance. Therefore, the excellent performance of BIPSPI is at least partially a result of structural similarity between parts of its training set and our test set. However, considering low similarity complexes with TM‐score <0.6, BIPSPI still yields the best performance between all the considered predictors (Figure S2, right panel), that is, overfitting is not the only factor causing this predictor superiority.

### 3.3 Docking with the constraints from the binding site predictions

**FIGURE 5:**
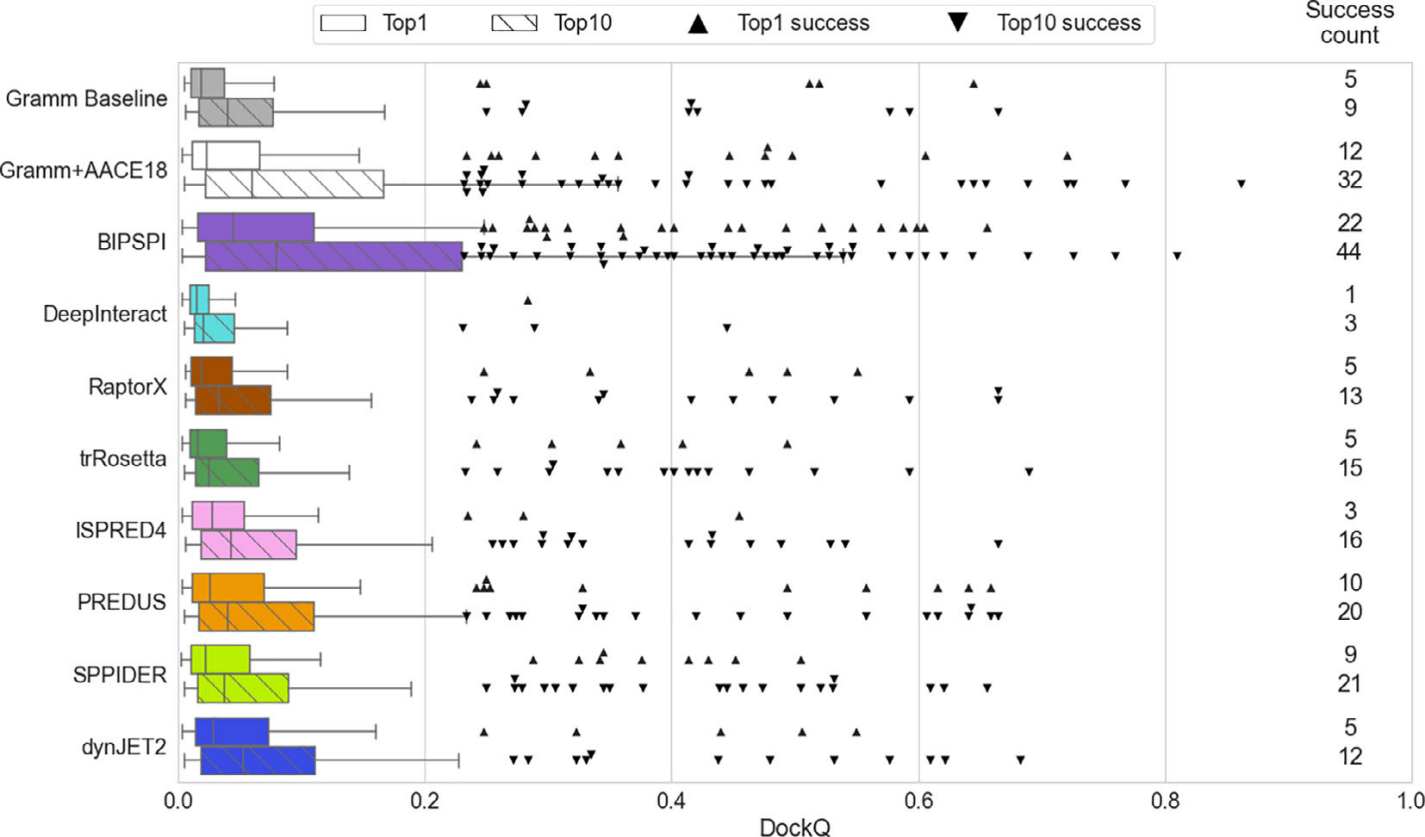
Performance of docking with constraints obtained by different interface predictors on 220 binary complexes from DOCKGROUND benchmark set 4. Horizontal bars represent the box‐and‐whiskers distributions of the DockQ scores, and each point represents a successful docking model (DockQ >0.23). Nonstriped bars and upward‐pointing triangles display results obtained for the top‐ranked docking models, while striped bars and downward‐pointing triangles pertain to the model with the best DockQ score among the top 10 docking models. Pairs of success counts represent the number of targets for which successful docking models were generated in the corresponding docking run within top 1 (upper number) and top 10 (lower number) docking models

Next, we examined the ability to use the interface predictions to score docking models. Docking models from the GRAMM scan stage (GRAMM baseline) were re‐scored using the interface probabilities (Equation 1) from the interface predictors listed in Table 1. We have also considered docking models re‐scored by the AACE18 potential^43^ for comparison. A summary of the results is shown in Figure 5 and Table S2. The most near‐native docking models are top‐ranked using the BIPSPI predictions, reaching SR(10) ~25% and SR(1) ~13%. Rescoring with this interface predictor is better than using the AACE18 potential (SR(10) ~18% and SR(1) ~7%).

Re‐scoring the docking models with predictions from the other predictors does not significantly improve overall docking performance compared to the docking with scoring by shape complementarity only (GRAMM baseline in Figure 5), and they are far from the performance level of the AACE18. SPPIDER and PREDUS predictions yield a slight improvement over the baseline docking, while all the other tested methods do not provide any significant improvement. Comparative analysis of predictor‐driven docking reveals that PREDUS and SPPIDER move up near‐native docking models for a few different complexes respective to BIPSPI. Comparing interface predictors‐based scorings (Figure S3), only in one case (PDB 1nbf) 6 out of 8 predictor‐driven dockings brought an acceptable model to the top of the prediction list. Further, top‐1 acceptable docking models were obtained by four predictors only for three other complexes (PDBs 1b27, 1vrs, and 1yu6). Thus, although the general impact of most predictors is low, there is a certain degree of complementarity between some of them (BIPSPI, PREDUS and SPPIDER), and their joint utilization could enhance cumulative docking success significantly.

There are 12 complexes for which BIPSPI constraints failed to produce a top‐1 near‐native docking model while other interface predictors succeeded. Two of the complexes exhibit DockQ score <0.03 for the top‐ranked BIPSPI dockings (PDBs 2zae, 3pro). These “extreme” failures, together with one additional case (PDB 3bx1), are caused by a failed interface prediction of BIPSPI (Table S3). For all other cases, the BIPSPI overall interface prediction quality is comparable to the best other method or better. Thus, failures here seem to be caused by BIPSPI tendency to be very precise (high PPV) at the expense of prediction completeness (data not shown). This leads to the number of generated (although correct) interface constraints being too weak to avoid significant rotational freedom between the two interacting patches. Note that considering acceptable models from top‐10 docking models did not increase consistently the number of complexes for which constraints from the most predictors lead to the successful docking.

Finally, it should be noted that BIPSPI and AACE18 scoring complement each other. Only three near‐native top‐1 complexes are shared, while BIPSPI and AACE18 separately succeeded for another 19 and 9 complexes, respectively (Figure S3F). When considering near‐native docking models from the top 10 docking solutions, that overlap is slightly more considerable (14 common cases compared with 18 unique for AACE18 and 30 for BIPSPI).

### 3.4 Simulated predictions

**FIGURE 6:**
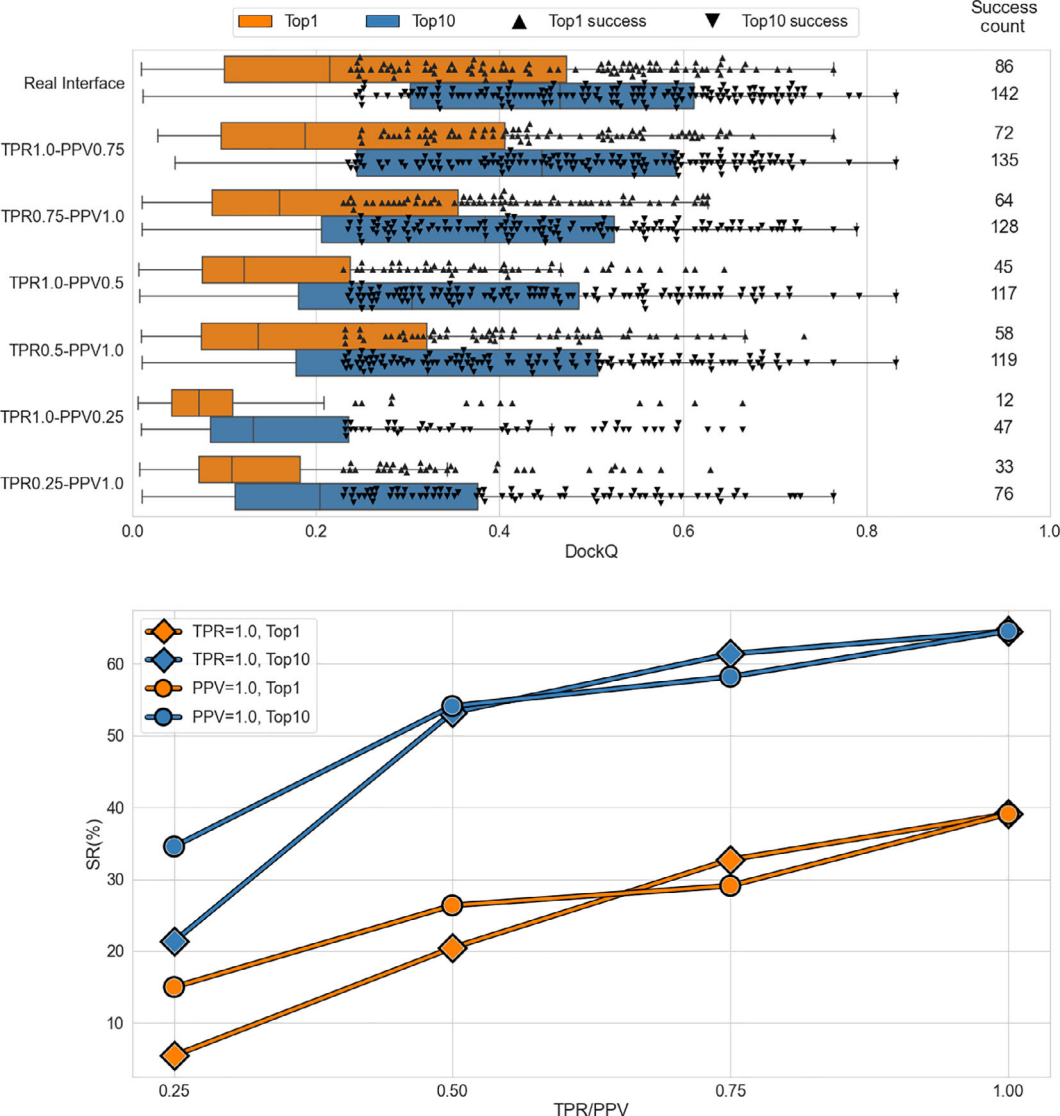
Performance of docking with constraints obtained from different simulated interfaces on 220 binary complexes from DOCKGROUND benchmark set 4. In the top panel, horizontal bars represent the box‐and‐whiskers distributions of the DockQ scores, and each point represents a successful docking model (DockQ >0.23). Orange bars and upward‐pointing triangles display results obtained for the top‐ranked docking models, while blue bars and downward‐pointing triangles pertain to the model with the best DockQ score among the top 10 docking models. Pairs of success counts represent the number of targets for which successful docking models were generated in the corresponding docking run within top 1 (upper number) and top 10 (lower number) docking models. The bottom panel displays success rates for top 1 (orange) and top 10 (blue) docking models obtained for a series of simulated interfaces with varying PPV (diamonds) or TRP (circles) while another parameter (TPR or PPV, respectively) is kept 1. Lines are guides for the eye

Various algorithms tested in this study produce interface predictions with TPR and PPV varying from protein to protein. Thus, in order to test the performance of the docking protocol in a controlled scenario (i.e., at predefined TPR and PPV values, which are the same for all complexes in the dataset), we introduce certain levels of “noise” into the native interface (see Section 2). We have added noise by reducing PPV, that is, adding false interface residue, and reducing TPR, that is, removing correct interface residues. Results are reported in Figure 6 (top panel) and Table S3.

In general, docking success is reduced by both under‐ (false negatives) or over‐ (false positives) interface predictions. In the scoring scheme used in the paper (Equation 1), the contribution of a large patch of true interface residues (covering the entire interface, TPR = 1) overweights the contribution from a small amount of wrongly predicted noninterface residues (PPV = 0.75). On the other hand, even relatively small under‐prediction of the interface (TRP = 0.75) gives rise to the undesired energetical “freedom” in the ligand placement even in the absence of wrongly predicted noninterface residues (PPV = 1). The trend is reversed when the level of “noise” at the predicted interfaces increases, and this behavior is the same for both top 1 and top 10 docking models.

### 3.5 Score set docking

**FIGURE 7:**
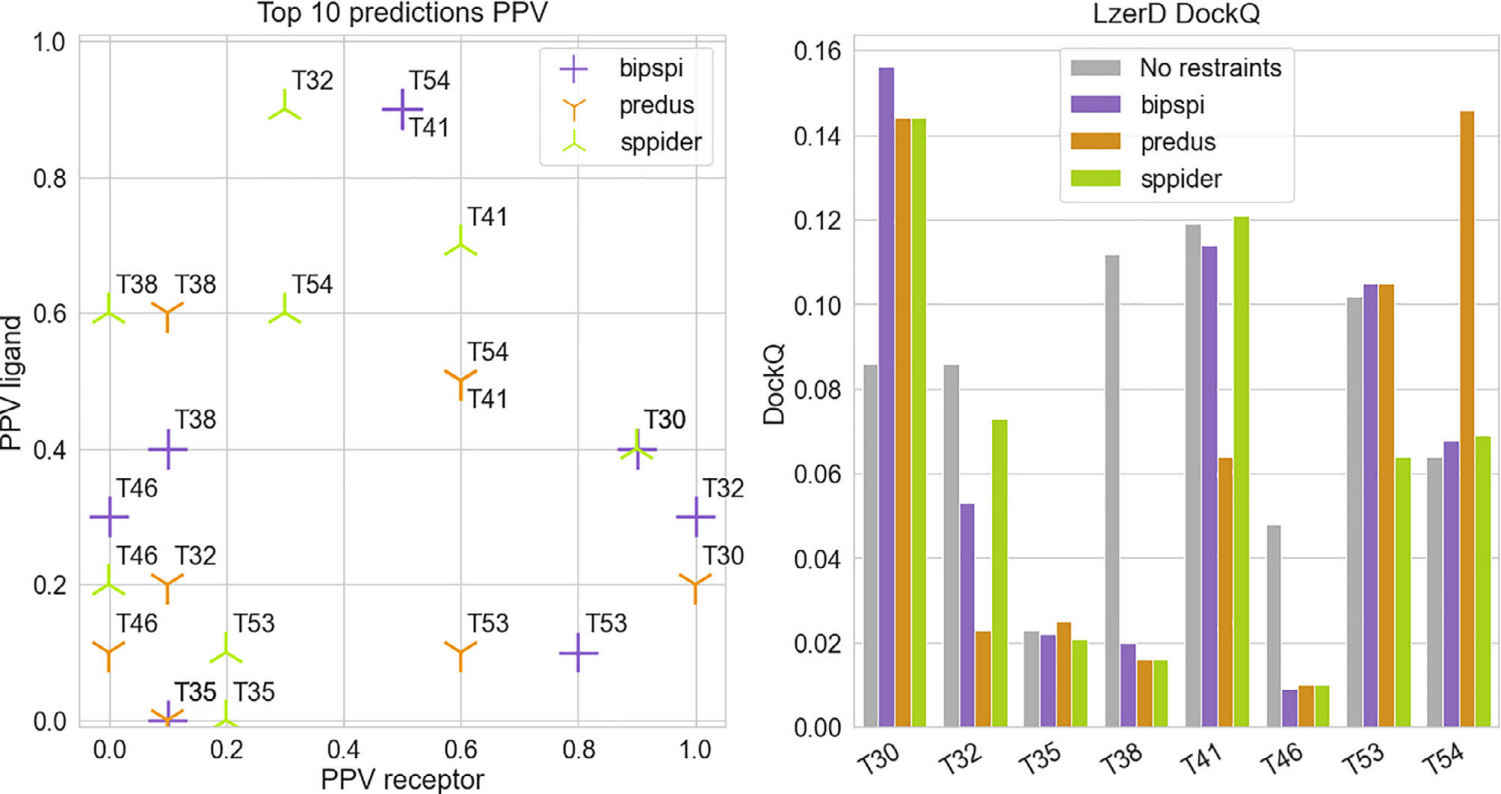
LzerD docking of CAPRI Score Set. Three interface predictors (BIPSPI, PREDUS, and SPPIDER) was used to generate restraints for the LzerD web server. (left) For each predictor, receptors precision values are compared to the respective ligand values. (right) DockQ scores represent for each complex the best among the top10 docking results selected from the LzerD web server using the mentioned restraints and no restraints at all

The LzerD web‐server has been adopted in combination with restraints generated from methods tested in this study. Interface predictions have been generated with BIPSPI, PREDUS, and SPPIDER for protein complexes from the CAPRI Score set. Restraints have been generated considering only the top 10 predictions for each interface prediction method, and protein chain restraints have been generated. We found that this number is sufficient in order to observe an effect on the docking result while at the same time allowing us to minimize the number of false positives. The precision of the obtained restraints varies widely, as shown in Figure 7. The most balanced high‐precision restraints were generated by SPPIDER for T41 (PDB code: 2wpt) with 6 and 7 correct restraints. Other predictions reach even higher numbers of correct restraints, but just for one of the two chains while having way lower PPV for the other one. All three methods obtain low‐quality restraints (PPV of both chains ≤0.4) for two targets (T35 and T46, PDB codes 2w5f and 3q87, respectively).

Remarkably, no docking models produced with the LzerD server possessed an “acceptable” DockQ score (Figure 7, right panel). Nevertheless, in two cases (T30 and T54, PDB codes 2rex and 4jw3 respectively), restraints improved the quality of the docking models. In the case of T30, improvements were achieved by all the tested interface predictors, while for T54, the DockQ score was only boosted by PREDUS. In other three cases (T35, T41, and T53, PDB codes 2w5f, 2wpt, and 4jw2, respectively), adding restraints did not produce any significant DockQ change, while PREDUS and SPPIDER restraints even reduced the quality of the docking models for T41 and T53, respectively. Finally, in the remaining three cases (T32, T38, and T46, PDB codes 3bx1, 3fm8, and 3q87, respectively) the docking without restraints yielded better models than the models generated using restraints.

Nevertheless, these results are limited by the small number of complexes. Interestingly, the number of cases where interface restraints are useful is surpassed by a comparable number of cases that may worsen the situation. Furthermore, the similarity between restraints precision clashes and the very different related docking outcomes (BIPSPI predictions for T30 and T32 and PREDUS predictions for T41 and T54 are very similar while the docking results are quite different). This observation supports the idea that more complex factors, like for instance restraints geometry relative to the real interface, are important to drive the correct choice of interface restraints.

### 3.6 Complex‐wise analysis

**FIGURE 8:**
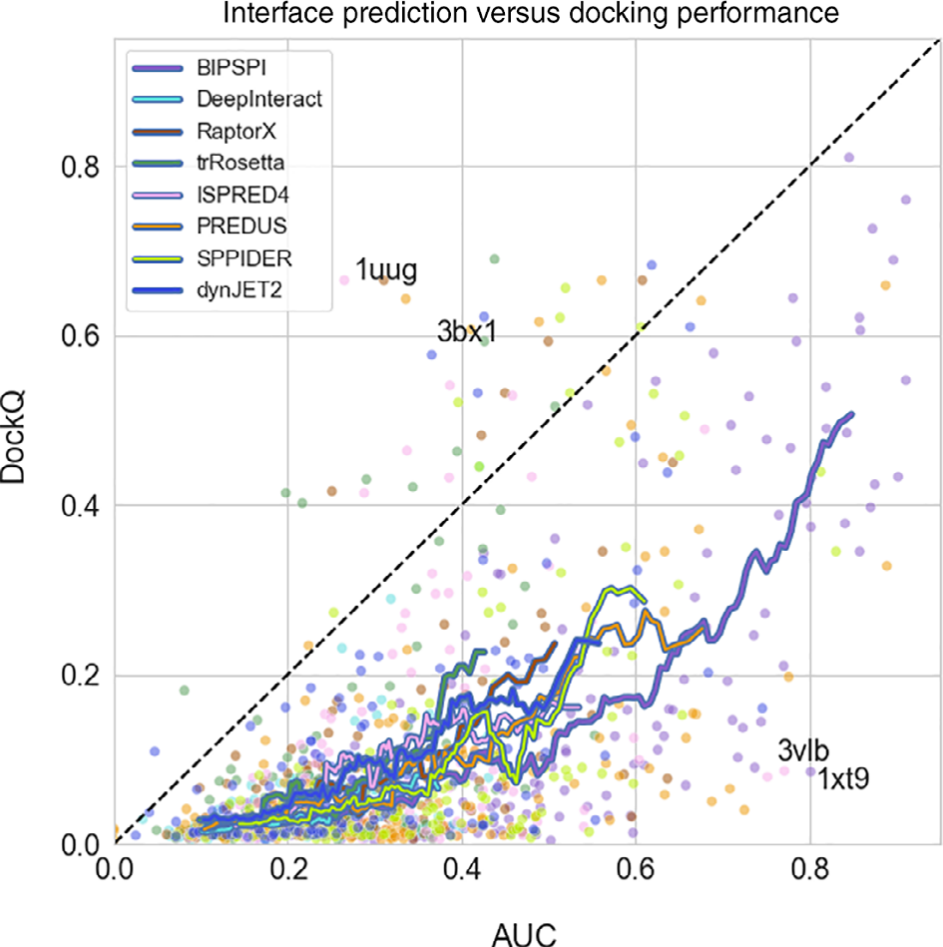
Correlation between area under precision‐recall curve (AUC) and the DockQ score of the best among top 10 docking models for various interface predictors. Data are shown only for those 177 binary complexes from DOCKGROUND benchmark set 4 with near‐native docking models in the upper baseline docking (constraints derived from the native interfaces). For each complex, averaged receptor and ligand's AUC are plotted. Running averages have been obtained from a sliding window of 20 data points

**FIGURE 9:**
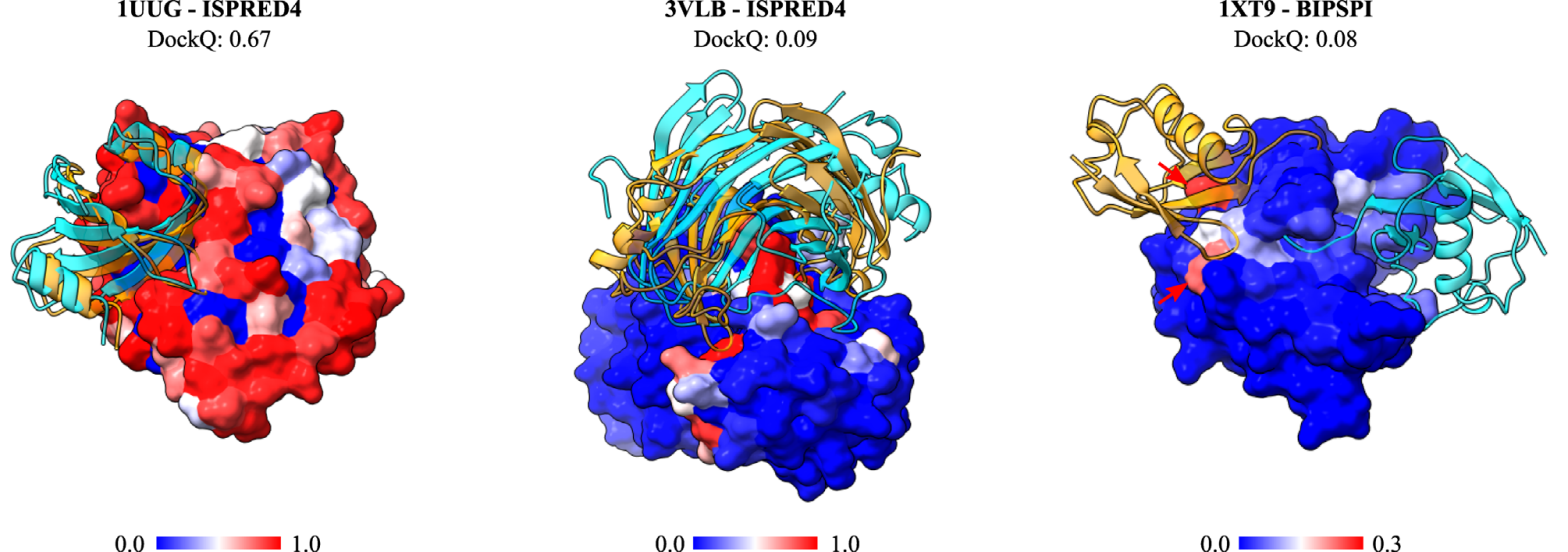
Three examples of constrained docking results; receptor is represented by atomic surfaces; all receptor atoms are colored according to the score given from the interface predictor, with blue to red transition indicating low to high scores; two ligands depicted in cartoon style are present in each structure, one in the native position colored in cyan and one resulting from the docking procedure (best in top 10 ranks), colored in orange; (left) acceptable docking compared to the bound structure with PDB ID 1uug yielding a DockQ score of 0.67, resulted from application of ISPRED4 scorings as constraints; (middle) example of incorrect docking due to rotational ambiguity in the relative orientation of the correct interfaces; docking produced using ISPRED4 scores (which yields a DockQ score of 0.09) is rotated of 180° around a vertical axis passing through the center of the receptor, respective to the native bound structure with PDB ID 3vlb; (right) example of incorrect docking due to high scoring of residues close but not belonging to the actual interface; the docking (with DockQ score of 0.02) is obtained adopting BIPSPI predictions and compared to the bound structure with PDB ID 1xt9; nevertheless the average 0.8 AUC value for this complex interface predictions, False Positive residues (indicated with red arrows) with high scoring are enough to drive the docking toward a completely wrong configuration. Those residues are part of the receptor catalytic site

The DockQ score for the docking models exhibits a strong correlation to the AUC of the interface predictors for the corresponding protein chains. (Figure 8). Few exceptions are found in complexes with high shape complementarity (Figure 9A), which is sufficient in some cases to achieve acceptable dockings even with low‐quality constraints. Another possibility to obtain good dockings from noisy constraints is the combination of wide scattering of false positives predictions over the entire surface and tightly packed true positives. Such scattering, observed, for instance, in dynJET2 predictions, allows in some cases successful docking regardless of somewhat inaccurate predictions (data for PDB 3bx1 in Table S3).

Constraint quality in our protocol also seems to be an essential but not sufficient condition for successful docking. A significant number of complexes exhibit low DockQ scores (~0.1) for the best out of the top 10 docking models, even with large AUC values (Figure 8). In those docking models, the ligand is placed into or close to the correct binding site of the receptor with the correct patches of ligand and receptor residues facing each other but with a wrong mutual orientation (Figure 9B). Subsequently, rotational freedom is quite a common pitfall of using interface constraints predicted independently for the receptor and ligand and can be seen as an intrinsic limitation of this method.

A fascinating case is given by the complex between the Den1 protease and Nedd8, a Ubiquitin‐like protein (Figure 9C). The biological role of this complex is to activate Nedd8 by removing a portion of its disordered C‐terminal.^53^ Predicting the interface of this complex with BIPSPI identifies a strong signal in the protease catalytic triad residues, shown by arrows in Figure 8C. These residues are located at the very edge of the interface and have better scores than the other predictions in the interface of Den1. The highest scores for the Nedd8 predictions are obtained for the amino acids in the middle of the Nedd8 interface. Since the peripheral of the Den 1 interface is located far away from the central part of its interface, docking poses with those high‐scored predictions facing each other and thus favored by the scoring scheme (Equation 1) are incorrect with the location of the ligand far away from its native position (Figure 8C).

## 4 CONCLUSIONS

In this work, we analyzed the use of predicted interface residues for scoring template free docking solutions. First, we show that interface information is sufficient to correctly identify an acceptable model for the vast majority of all targets that could be generated. Using predictions derived from interface and contact predictors, we found that one predictor, BIPSPI, was superior to all the other tested ones. Using the interfaces predicted from BIPSPI, almost twice (13% vs. 7%) as many first ranked models are acceptable (DockQ >0.23). However, when applied to a more complex docking scenario, BIPSPI did not show any increased performance respective to other tested predictors, leaving open questions on how a method to generate docking constraints should be properly evaluated. Further, the methodology used in this paper can be applied to evaluate other interface or contact prediction methods, thanks to its simplicity and flexibility.

### PEER REVIEW

The peer review history for this article is available at https://publons.com/publon/10.1002/prot.26330.

## Supporting information


**Appendix** S1: Supporting Information

## Data Availability

The data that support the findings of this study are openly available in Figshare at https://doi.org/10.17044/scilifelab.16438206.v1.

## References

[1] Panchenko A , Przytycka TM . Protein‐Protein Interactions and Networks: Identification, Computer Analysis, and Prediction. Springer Science & Business Media; 2010.

[2] Fu H . Protein‐Protein Interactions: Methods and Applications. Springer Science & Business Media; 2004.

[3] Anishchenko I , Kundrotas PJ , Vakser IA . Modeling complexes of modeled proteins. Proteins. 2017;85:470‐478. 27701777 10.1002/prot.25183 PMC5313347

[4] Keskin O , Gursoy A , Ma B , Nussinov R . Principles of protein‐protein interactions: what are the preferred ways for proteins to interact? Chem Rev. 2008;108:1225‐1244. 18355092 10.1021/cr040409x

[5] Nooren IMA . Diversity of protein‐protein interactions. EMBO J. 2003;22:3486‐3492. 12853464 10.1093/emboj/cdg359 PMC165629

[6] Maleki, M , Aziz, MM , Rueda, L . Analysis of relevant physicochemical properties in obligate and non‐obligate protein‐protein interactions. 2011 IEEE International Conference on Bioinformatics and Biomedicine Workshops (BIBMW); 2011. doi:10.1109/bibmw.2011.6112397

[7] Pierce BG , Wiehe K , Hwang H , Kim BH , Vreven T , Weng Z . ZDOCK server: interactive docking prediction of protein‐protein complexes and symmetric multimers. Bioinformatics. 2014;30:1771‐1773. 24532726 10.1093/bioinformatics/btu097 PMC4058926

[8] Moal IH , Chaleil RAG , Bates PA . Flexible protein‐protein docking with SwarmDock. Methods Mol Biol. 2018;1764:413‐428. 29605931 10.1007/978-1-4939-7759-8_27

[9] Andrusier N , Mashiach E , Nussinov R , Wolfson HJ . Principles of flexible protein‐protein docking. Proteins. 2008;73:271‐289. 18655061 10.1002/prot.22170 PMC2574623

[10] Moal IH , Torchala M , Bates PA , Fernández‐Recio J . The scoring of poses in protein‐protein docking: current capabilities and future directions. BMC Bioinform. 2013;14:286. 10.1186/1471-2105-14-286 PMC3850738 24079540

[11] Wang X , Flannery ST , Kihara D . Protein docking model evaluation by graph neural networks. Front Mol Biosci. 2021;8:647915. 34113650 10.3389/fmolb.2021.647915 PMC8185212

[12] Vreven T , Moal IH , Vangone A , et al. Updates to the integrated protein–protein interaction benchmarks: docking benchmark version 5 and affinity benchmark version 2. J Mol Biol. 2015;427:3031‐3041. 26231283 10.1016/j.jmb.2015.07.016 PMC4677049

[13] Porter KA , Desta I , Kozakov D , Vajda S . What method to use for protein–protein docking? Curr Opin Struct Biol. 2019;55:1‐7. 30711743 10.1016/j.sbi.2018.12.010 PMC6669123

[14] Andreani J , Quignot C , Guerois R . Structural prediction of protein interactions and docking using conservation and coevolution. WIREs Comp Mol Sci. 2020;10:e1470.

[15] Krippahl L , Barahona P . Protein docking with predicted constraints. Algorithms Mol Biol. 2015;10:9. 25722738 10.1186/s13015-015-0036-6 PMC4340843

[16] Zeng M , Zhang F , Wu F‐X , Li Y , Wang J , Li M . Protein‐protein interaction site prediction through combining local and global features with deep neural networks. Bioinformatics. 2020;36:1114‐1120. 31593229 10.1093/bioinformatics/btz699

[17] Northey TC , Barešić A , Martin ACR . IntPred: a structure‐based predictor of protein–protein interaction sites. Bioinformatics. 2018;34:223‐229. 28968673 10.1093/bioinformatics/btx585 PMC5860208

[18] Jiao X , Ranganathan S . Prediction of interface residue based on the features of residue interaction network. J Theor Biol. 2017;432:49‐54. 28818468 10.1016/j.jtbi.2017.08.014

[19] Sanchez‐Garcia R , Sorzano COS , Carazo JM , Segura J . BIPSPI: a method for the prediction of partner‐specific protein‐protein interfaces. Bioinformatics. 2019;35:470‐477. 30020406 10.1093/bioinformatics/bty647 PMC6361243

[20] Vajdi A , Zarringhalam K , Haspel N . Patch‐DCA: improved protein interface prediction by utilizing structural information and clustering DCA scores. Bioinformatics. 2020;36:1460‐1467. 31621841 10.1093/bioinformatics/btz791

[21] Hou Q , De Geest PFG , Vranken WF , Heringa J , Feenstra KA . Seeing the trees through the forest: sequence‐based homo‐ and heteromeric protein‐protein interaction sites prediction using random forest. Bioinformatics. 2017;33:1479‐1487. 28073761 10.1093/bioinformatics/btx005

[22] Deng A , Zhang H , Wang W , et al. Developing computational model to predict protein‐protein interaction sites based on the XGBoost algorithm. Int J Mol Sci. 2020;21:2274. 10.3390/ijms21072274 PMC7178137 32218345

[23] Daberdaku S , Ferrari C . Exploring the potential of 3D Zernike descriptors and SVM for protein–protein interface prediction. BMC Bioinform. 2018;19:35. 10.1186/s12859-018-2043-3 PMC5802066 29409446

[24] Zeng H , Wang S , Zhou T , et al. ComplexContact: a web server for inter‐protein contact prediction using deep learning. Nucleic Acids Res. 2018;46:W432‐W437. 29790960 10.1093/nar/gky420 PMC6030867

[25] Yang J , Anishchenko I , Park H , Peng Z , Ovchinnikov S , Baker D . Improved protein structure prediction using predicted interresidue orientations. Proc Natl Acad Sci U S A. 2020;117:1496‐1503. 31896580 10.1073/pnas.1914677117 PMC6983395

[26] Savojardo C , Fariselli P , Martelli PL , Casadio R . ISPRED4: interaction sites PREDiction in protein structures with a refining grammar model. Bioinformatics. 2017;33:1656‐1663. 28130235 10.1093/bioinformatics/btx044

[27] Fernández‐Recio J . Prediction of protein binding sites and hot spots. WIREs Comput Mol Sci. 2011;1:680‐698.

[28] Zhou, T.‐M. , Wang, S. & Xu, J . Deep learning reveals many more inter‐protein residue‐residue contacts than direct coupling analysis doi:10.1101/240754

[29] Ovchinnikov S , Kamisetty H , Baker D . Robust and accurate prediction of residue–residue interactions across protein interfaces using evolutionary information. Elife. 2014;3:e02030. 24842992 10.7554/eLife.02030 PMC4034769

[30] Sotiropoulos DN , Tsihrintzis GA . The class imbalance problem. Machine Learning Paradigms. Springer; 2017:51‐78.

[31] Xue LC , Jordan RA , El‐Manzalawy Y , Dobbs D , Honavar V . DockRank: ranking docked conformations using partner‐specific sequence homology‐based protein interface prediction. Proteins. 2014;82:250‐267. 23873600 10.1002/prot.24370 PMC4417613

[32] Li B , Kihara D . Protein docking prediction using predicted protein‐protein interface. BMC Bioinform. 2012;13:7. 10.1186/1471-2105-13-7 PMC3287255 22233443

[33] Schneider S , Zacharias M . Scoring optimisation of unbound protein‐protein docking including protein binding site predictions. J Mol Recognit. 2012;25:15‐23. 22213447 10.1002/jmr.1165

[34] de Vries SJ , Bonvin AMJJ . CPORT: a consensus interface predictor and its performance in prediction‐driven docking with HADDOCK. PLoS One. 2011;6:e17695. 21464987 10.1371/journal.pone.0017695 PMC3064578

[35] Vakser IA . Evaluation of GRAMM low‐resolution docking methodology on the hemagglutinin‐antibody complex. Proteins. 1997;1(Suppl):226‐230. 9485517

[36] Kundrotas PJ , Anishchenko I , Dauzhenka T , et al. Dockground: a comprehensive data resource for modeling of protein complexes. Protein Sci. 2018;27:172‐181. 28891124 10.1002/pro.3295 PMC5734278

[37] Fu L , Niu B , Zhu Z , Wu S , Li W . CD‐HIT: accelerated for clustering the next‐generation sequencing data. Bioinformatics. 2012;28:3150‐3152. 23060610 10.1093/bioinformatics/bts565 PMC3516142

[38] Henikoff S , Henikoff JG . Performance evaluation of amino acid substitution matrices. Proteins. 1993;17:49‐61. 8234244 10.1002/prot.340170108

[39] Hwang H , Pierce B , Mintseris J , Janin J , Weng Z . Protein‐protein docking benchmark version 3.0. Proteins. 2008;73:705‐709. 18491384 10.1002/prot.22106 PMC2726780

[40] Lensink MF , Wodak SJ . Score_set: a CAPRI benchmark for scoring protein complexes. Proteins. 2014;82:3163‐3169. 25179222 10.1002/prot.24678

[41] Kozakov D , Hall DR , Xia B , et al. The ClusPro web server for protein–protein docking. Nat Protoc. 2017;12:255‐278. 28079879 10.1038/nprot.2016.169 PMC5540229

[42] Sinha R , Kundrotas PJ , Vakser IA . Protein docking by the interface structure similarity: how much structure is needed? PLoS One. 2012;7:e31349. 22348074 10.1371/journal.pone.0031349 PMC3278447

[43] Anishchenko I , Kundrotas PJ , Vakser IA . Contact potential for structure prediction of proteins and protein complexes from Potts model. Biophys J. 2018;115:809‐821. 30122295 10.1016/j.bpj.2018.07.035 PMC6127504

[44] Christoffer C , Chen S , Bharadwaj V , et al. LZerD webserver for pairwise and multiple protein‐protein docking. Nucleic Acids Res. 2021;49:W359‐W365. 33963854 10.1093/nar/gkab336 PMC8262708

[45] Porollo A , Meller J . Prediction‐based fingerprints of protein‐protein interactions. Proteins. 2007;66:630‐645. 17152079 10.1002/prot.21248

[46] Zhang QC , Deng L , Fisher M , Guan J , Honig B , Petrey D . PredUs: a web server for predicting protein interfaces using structural neighbors. Nucleic Acids Res. 2011;39:W283‐W287. 21609948 10.1093/nar/gkr311 PMC3125747

[47] Dequeker C , Laine E , Carbone A . Decrypting protein surfaces by combining evolution, geometry, and molecular docking. Proteins. 2019;87:952‐965. 31199528 10.1002/prot.25757 PMC6852240

[48] Morehead, A , Chen, C , Cheng, J . Geometric transformers for protein interface contact prediction. *arXiv [cs.LG]*; 2021.

[49] Pozzati G , Zhu W , Bassot C , Lamb J , Kundrotas P , Elofsson A . Limits and potential of combined folding and docking. Bioinformatics. 2021;38:954‐961. doi:10.1093/bioinformatics/btab760 PMC8796369 34788800

[50] Kabsch W , Sander C . Dictionary of protein secondary structure: pattern recognition of hydrogen‐bonded and geometrical features. Biopolymers. 1983;22:2577‐2637. 6667333 10.1002/bip.360221211

[51] Basu S , Wallner B . DockQ: a quality measure for protein‐protein docking models. PLoS One. 2016;11:e0161879. 27560519 10.1371/journal.pone.0161879 PMC4999177

[52] Janin J , Henrick K , Moult J , et al. CAPRI: a critical assessment of PRedicted interactions. Proteins: Struct Funct Genet. 2003;52:2‐9. 12784359 10.1002/prot.10381

[53] Reverter D , Wu K , Erdene TG , Pan ZQ , Wilkinson KD , Lima CD . Structure of a complex between Nedd8 and the Ulp/Senp protease family member Den1. J Mol Biol. 2005;345:141‐151. 15567417 10.1016/j.jmb.2004.10.022

