# Supplementary material for: Scoring of protein–protein docking models utilizing predicted interface residues
Source: Proteins. 2022 Mar 14;90(7):1493–505. doi: 10.1002/prot.26330 (PMC9314140; doi:10.1002/prot.26330)
Supplement: Supplementary file 1 — Appendix S1: Supporting Information [file PROT-90-1493-s001.pdf]

SUPPLEMENTARY MATERIAL

Table S1. Summary of PDB and chain codes for targets selected from the CAPRI Score_set.

CAPRI code	Bound PDB	Unbound receptor PDB	Unbound ligand PDB
T30	2REX_A:B	2CLS_A	2R2O_A
T32	3BX1_A:C	1SVN_A	1AVA_C
T35	2W5F_A:A	1N82_A	1DYO_A
T38	3FM8_A:C	3FEH_A	2G1L_A
T41	2WPT_A:B	1FSJ_B	2NO8_1
T46	3Q87_A:B	1P91_A	2J6A_A
T53	4JW2_A:B	3LTJ_A	3LTJ_A
T54	4JW3_A:C	3LTJ_A	2CBO_A

Table S2. Summary of the docking results with constraints derived from the various interface predictors.

Docking	Top 1				Top 10			
	Median	Mean	St.Dev	SR(1),%	Median	Mean	St.Dev	SR(10), %
Gramm Baseline	0.02	0.04	0.08	2.9	0.04	0.07	0.10	5.1
Gramm + AACE18	0.02	0.07	0.11	6.9	0.06	0.13	0.17	18.3
BIPSPI	0.04	0.10	0.14	12.6	0.08	0.17	0.19	25.1
DeepInteract	0.01	0.03	0.03	0.6	0.02	0.04	0.05	1.7
RaptorX	0.02	0.04	0.07	2.9	0.03	0.08	0.12	7.4
TrRosetta	0.02	0.04	0.07	2.9	0.03	0.07	0.12	8.6
ISPRED4	0.03	0.04	0.05	1.7	0.04	0.09	0.12	9.1
PREDUS	0.03	0.06	0.11	5.7	0.04	0.10	0.14	11.4
SPPIDER	0.02	0.05	0.09	5.1	0.04	0.09	0.14	12.0
DynJET2	0.03	0.06	0.08	2.9	0.05	0.09	0.12	6.9

Table S3. Summary of the docking results with constraints derived from the simulated interfaces.

Docking	Top 1				Top 10			
	Median	Mean	St.Dev	SR(1), %	Median	Mean	St.Dev	SR(10), %
Real Interface	0.21	0.28	0.20	49.1	0.47	0.45	0.20	81.1
TPR1.0 PPV0.75	0.19	0.25	0.19	41.1	0.45	0.42	0.20	77.1
TPR0.75 PPV1.0	0.16	0.22	0.17	36.6	0.38	0.37	0.20	73.1
TPR1.0 PPV0.5	0.12	0.18	0.14	25.7	0.30	0.34	0.19	66.9
TPR0.5 PPV1.0	0.14	0.21	0.17	33.1	0.32	0.35	0.20	68.0
TPR1.0 PPV0.25	0.07	0.10	0.10	6.9	0.13	0.19	0.16	26.9
TPR0.25 PPV1.0	0.11	0.15	0.12	18.9	0.20	0.26	0.19	43.4

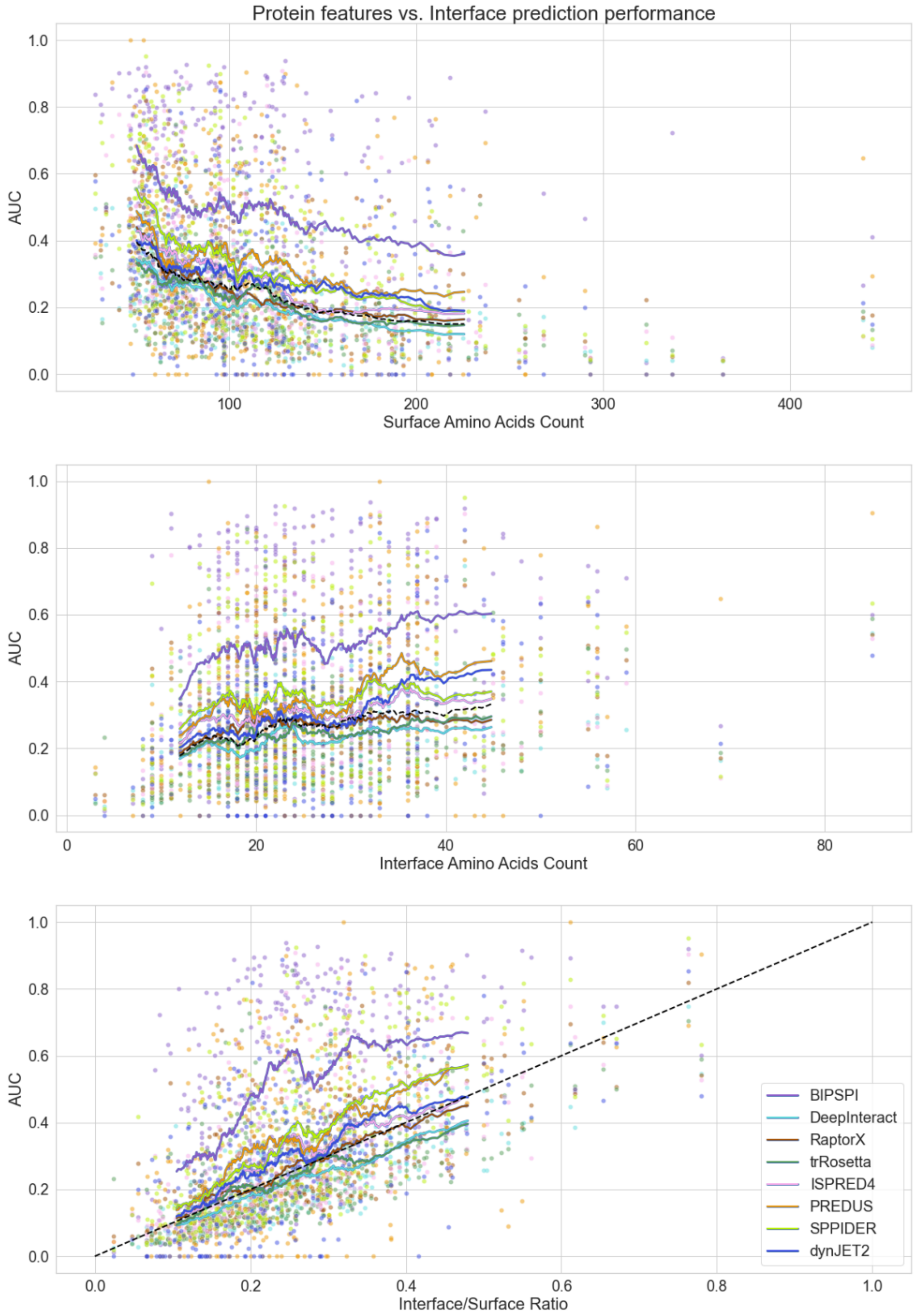

Figure S1. *Impact of single proteins features over interface prediction performance.* Each point represents a protein from the DOCKGROUND dataset, colored accordingly to the predictor adopted to obtain interface estimates. Running averages have been calculated in both cases considering a window of 50 proteins sliding along the independent variable axis. Dashed lines indicate the expected random predictor performance in terms of interface to surface residues ratio. In the left panel, the number of surface residues in single proteins (determined by relative solvent accessibility value > 0.2) has been plotted against the related interface prediction AUC for all considered predictors. In the middle panel, the number of residues which belong to the native complex interface has been plotted instead against AUC values. Finally, in the right panel, the ratio between the number of surface residues and the number of residues which are part of the interface has been plotted as well, against the interface prediction AUC.

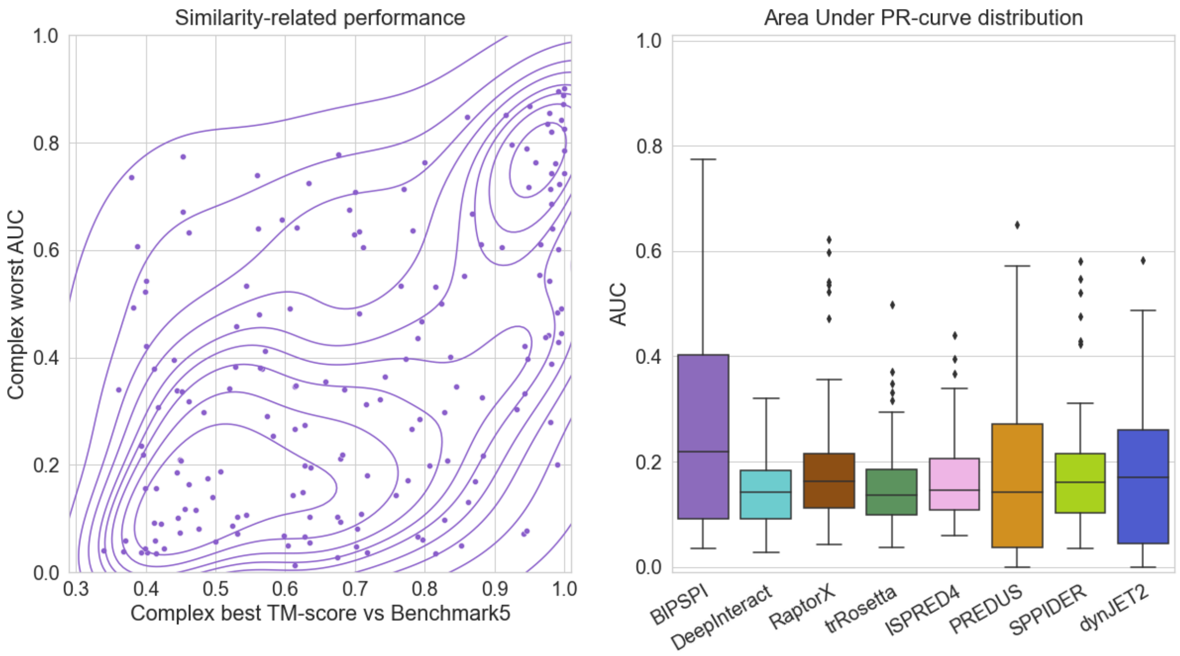

Figure S2. *Correlation of interface prediction performance to BIPSPI training set similarity.* Left panel shows the correlation between the highest TM-score obtained comparing DOCKGROUND and Benchmark5 complexes and the worst chain predictions generated by BIPSPI for the same complexes. Kernel density estimates lines provide a visual help to define point density. The right panel reports distributions, for each predictor, of each complex worst chain prediction, for each complex with TM-score against Benchmark5 lower than 0.6.

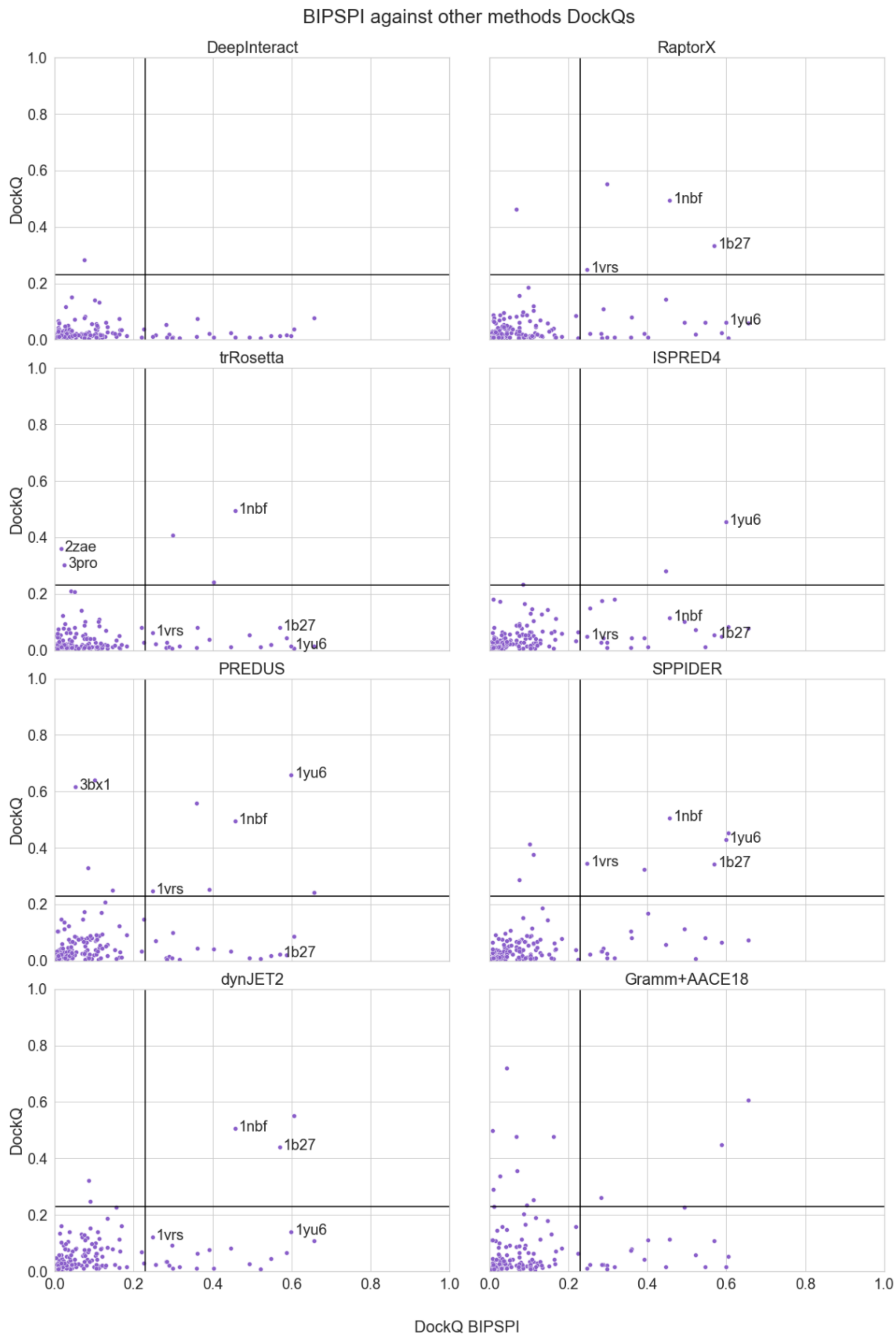

Figure S3. *Correlation of BIPSPI and other predictors top ranked docking results.* Scatterplot showing for each complex the top ranked docking DockQ scores obtained using BIPSPI constraints, versus the scores obtained using other predictors constraints. Particular docking cases with acceptable quality (DockQ score >= 0.23) with at least one predictor have been annotated with the respective PDB ID. Horizontal and vertical lines indicate the thresholds to consider a docking acceptable.

Table S3. Summary of the docking results and predictions AUC for individual complexes

Complex	Scoring	Receptor AUC	Ligand AUC	DockQ (Top1)	DockQ (Top10)
1uug	BIPSPI	**0.85**	**0.89**	**0.66**	**0.73**
	Deepinteract	0.45	0.41	0.08	0.08
	RaptorX	0.17	0.45	0.06	0.67
	TrRosetta	0.16	0.51	0.01	0.02
	ISPRED4	0.24	0.29	0.08	0.67
	PREDUS	0.71	0.41	0.24	0.67
	SPPIDER	0.41	0.64	0.07	0.08
	DynJET2	0.43	0.42	0.11	0.62
	AACE	/	/	0.61	0.66
1xt9	BIPSPI	0.71	0.89	0.09	0.09
	Deepinteract	0.40	0.45	0.02	0.03
	RaptorX	0.40	0.69	0.10	0.15
	TrRosetta	0.31	0.63	0.02	0.07
	ISPRED4	0.25	0.75	0.23	0.33
	PREDUS	**0.78**	**1.00**	**0.33**	0.33
	SPPIDER	0.48	0.83	0.15	0.15
	DynJET2	0.63	0.64	0.07	**0.44**
	AACE	/	/	0.02	0.15
2zae	BIPSPI	0.16	**0.73**	0.02	0.02
	Deepinteract	0.15	0.37	0.03	0.05
	RaptorX	0.69	0.60	0.03	0.45
	TrRosetta	**0.86**	0.16	**0.36**	**0.52**
	ISPRED4	0.23	0.25	0.04	0.12
	PREDUS	0.34	0.30	0.15	0.15
	SPPIDER	0.17	0.38	0.02	0.02
	DynJET2	0.18	0.40	0.10	0.14
	AACE	/	/	0.02	0.02

Bold values are the highest ones for each complex column. Table continues on the next page.

3bx1	BIPSPI	**0.80**	0.17	0.05	0.06
	Deepinteract	0.12	0.10	0.01	0.01
	RaptorX	0.14	0.14	0.04	0.01
	TrRosetta	0.12	0.12	0.01	0.01
	ISPRED4	0.22	0.27	0.06	0.06
	PREDUS	0.44	**0.53**	**0.62**	**0.62**
	SPPIDER	0.43	0.22	0.07	0.10
	DynJET2	0.30	0.43	0.08	0.58
	AACE	/	/	0.09	0.20
3pro	BIPSPI	0.32	**0.59**	0.03	0.03
	Deepinteract	0.28	0.24	0.01	0.01
	RaptorX	0.24	0.45	0.03	0.06
	TrRosetta	**0.57**	0.28	**0.30**	**0.35**
	ISPRED4	0.41	0.36	0.01	0.03
	PREDUS	**0.57**	0.28	0.03	0.03
	SPPIDER	0.24	0.37	0.02	0.04
	DynJET2	0.19	0.50	0.02	0.02
	AACE	/	/	0.03	0.06
3vlb	BIPSPI	**0.83**	**0.89**	**0.52**	**0.61**
	Deepinteract	0.22	0.22	0.01	0.01
	RaptorX	0.16	0.17	0.02	0.02
	TrRosetta	0.13	0.37	0.01	0.01
	ISPRED4	0.64	0.86	0.07	0.08
	PREDUS	0.05	0.50	0.01	0.01
	SPPIDER	0.13	0.38	0.01	0.01
	DynJET2	0.10	0.42	0.01	0.02
	AACE	/	/	0.06	0.06

Bold values are the highest ones for each complex column.
